# Characterization of the complete chloroplast genome of *Musella lasiocarpa*

**DOI:** 10.1080/23802359.2018.1456367

**Published:** 2018-07-05

**Authors:** Lei Zhang, Xinyi Guo, Zefu Wang, Mingcheng Wang, Quanjun Hu

**Affiliations:** Key Laboratory for Bio-resource and Eco-environment of Ministry of Education, College of Life Sciences, Sichuan University, Chengdu, China

**Keywords:** *Musella lasiocarpa*, Musaceae, chloroplast genome

## Abstract

The whole chloroplast (cp) genome sequence of *Musella lasiocarpa* has been characterized from Illumina pair-end sequencing. The complete cp genome was 169,178 bp in length, containing a large single copy (LSC) region of 87,884 bp and a small single copy (SSC) region of 11,144 bp, which were separated by a pair of 35,075 bp inverted repeat (IR) regions. The genome contained 138 genes, including 88 protein-coding genes (87 PCG species), 37 tRNA genes (30 tRNA species), and eight ribosomal RNA genes (four rRNA species). The most of gene species occur as a single copy, while 23 gene species occur in double copies. The overall AT content of *M. lasiocarpa* cp genome is 63.3%, while the corresponding values of the LSC, SSC, and IR regions are 64.9, 69.2, and 60.3%, respectively. The cp genome sequence is similar to that of the genus Musa.

*Musella lasiocarpa* (Franch.) C. Y. Wu ex H. W. Li, a medicinal plant, is the only species in the monotypic genus *Musella* of the Musaceae. It is distributed in Guizhou, Sichuan, and Yunnan provinces in China. The fresh flowers and bracts can be used for medicine to stop bleeding and counteract inflammation. This medicinal plant is widely used to treat enteritis, constipation and female diseases, detoxify monkshood poisoning and alleviate drunkenness (Liu and Kress 2003). However, due to anthropogenic over-exploitation and decreasing distributions, this species needs urgent conservation. Knowledge of the genetic information of this species would contribute to the formulation of protection strategy. In this study, we assembled and characterized the complete chloroplast (cp) genome sequence of *M. lasiocarpa* based on the Illumina pair-end sequencing data (Figure 1).

**Figure 1.**
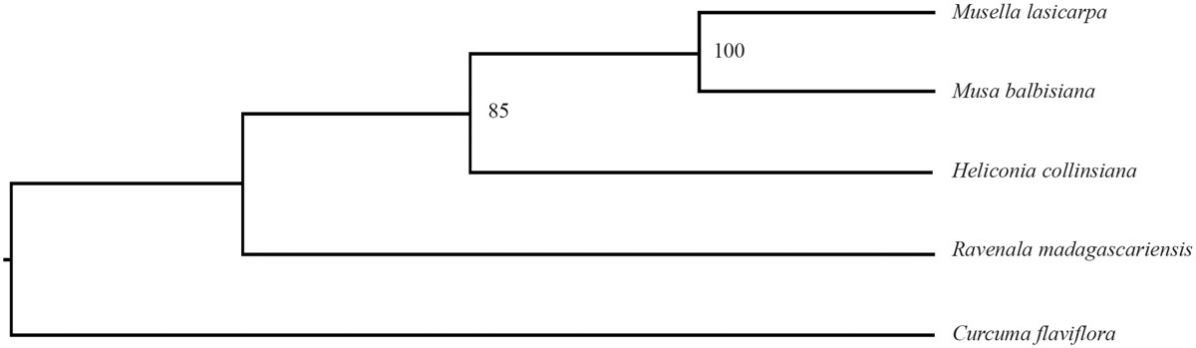
Phylogenetic relationships of Musaceae species using whole chloroplast genome. GenBank accession numbers: *C. flaviflora* (Nc_028729.1), *M. balbisiana* (Nc_028439.1), *H. collinsiana* (Nc_020362.1), and *R. madagascariensis* (Nc_022927.1).

Fresh leaves of *M. lasiocarpa* were collected from Dayao (Chuxiong, Yunnan, China; coordinates: 101°14′33″E, 25°54′10″N). Total genomic DNA was extracted with a modified CTAB method (Doyle and Doyle 1987). First, we obtained 10 million high quality pair-end reads for *M. lasiocarpa* and retained cp genome reads by mapping reads to all published Solanaceae cp genomes using BWA v0.7.12 (Li and Durbin 2009) and SAMtools v1.2 (Li et al. 2009). Second, we assembled these reads into a complete cp genome using Velvet v1.2.07 and Geneious v8.1.4 (Zerbino and Birney 2008; Kearse et al. 2012). Third, we annotated the plastid genomes using Plann v1.1 (Huang and Cronk 2015) and corrected the annotation with Geneious v8.1.4 (Kearse et al. 2012) and Sequin v13.70 (http://www.ncbi.nlm.nih.gov/Sequin/). A neighbour-joining (NJ) tree with 100 bootstrap replicates was inferred using TreeBeST 1.9.2 (Albert et al. 2009). The complete cp genome sequence was deposited in GenBank under accession number KY807173.

The *M. lasiocarpa* cp genome is 169,178 bp in length, exhibits a typical quadripartite structural organization, consisting of a large single copy (LSC) region of 87,884 bp, two inverted repeat (IR) regions of 35,075 bp and a small single copy (SSC) region of 11,144 bp. The cp genome contains 138 complete genes, including 88 protein-coding genes (87 PCGs), eight ribosomal RNA genes (four rRNAs), and 37 tRNA genes (30 tRNAs). Most genes occur in a single copy, while 23 genes occur in double, including all rRNAs (4.5S, 5S, 16S, and 23S rRNA), seven tRNAs (trnA-UGC, trnI-CAU, trnI-GAU, trnL-CAA, trnN-GUU, trnR-ACG, and trnV-GAC), and 12 PCGs (rps7, rps12, rps15, rps19, rpl2, rpl23, ndhA, ndhB, ndhH, ycf1, ycf15, and ycf68). The overall AT content of cp DNA is 63.3%, while the corresponding values of the LSC, SSC, and IR regions are 64.9%, 69.2%, and 60.3%, respectively. *Curcuma flaviflora* was used as an outgroup, phylogenetic analysis of three plastid genomes from published species of Solanaceae indicated that the *M. lasiocarpa* clustered together with *Musa balbisiana*, and then formed one clade with *Heliconia collinsiana* in the Musaceae.

In summary, the complete cp genome from this study not only provides important insight into conservation and restoration efforts for *M. lasiocarpa*, but also plays a critical role in constructing phylogeny of the Musaceae family.
